# Cautions about the reliability of pairwise gene correlations based on expression data

**DOI:** 10.3389/fmicb.2015.00650

**Published:** 2015-06-26

**Authors:** Scott Powers, Matt DeJongh, Aaron A. Best, Nathan L. Tintle

**Affiliations:** ^1^Department of Statistics, Stanford UniversityStanford, CA, USA; ^2^Department of Computer Science, Hope CollegeHolland, MI, USA; ^3^Department of Biology, Hope CollegeHolland, MI, USA; ^4^Department of Mathematics, Statistics and Computer Science, Dordt CollegeSioux Center, IA, USA

**Keywords:** Pearson correlation, operon prediction, mutual information, regulatory network inference, co-regulation

## Abstract

**Background:** Rapid growth in the availability of genome-wide transcript abundance levels through gene expression microarrays and RNAseq promises to provide deep biological insights into the complex, genome-wide transcriptional behavior of single-celled organisms. However, this promise has not yet been fully realized.

**Results:** We find that computation of pairwise gene associations (correlation; mutual information) across a set of 2782 total genome-wide expression samples from six diverse bacteria produces unexpectedly large variation in estimates of pairwise gene association—regardless of the metric used, the organism under study, or the number and source of the samples. We pinpoint the cause to sampling bias. In particular, in repositories of expression data (e.g., Gene Expression Omnibus, GEO), many individual genes show small differences in absolute gene expression levels across the set of samples. We demonstrate that these small differences are due mainly to “noise” instead of “signal” attributable to environmental or genetic perturbations. We show that downstream analysis using gene expression levels of genes with small differences yields biased estimates of pairwise association.

**Conclusions:** We propose flagging genes with small differences in absolute, RMA-normalized, expression levels (e.g., standard deviation less than 0.5), as potentially yielding biased pairwise association metrics. This strategy has the potential to substantially improve the confidence in genome-wide conclusions about transcriptional behavior in bacterial organisms. Further work is needed to further refine strategies to identify genes with small difference in expression levels prior to computing gene-gene association metrics.

## Introduction

The number of experimental conditions for which genome-wide gene expression data is available is rapidly increasing for all bacteria. This increase is due to maturing microarray technology and the advent of RNAseq technology, which has made measurement of gene expression possible for all bacteria, instead of only those for which pre-existing technology (e.g., custom microarrays) has existed.

While downstream analysis of gene expression data can take many forms, in numerous applications researchers wish to investigate whether statistical association (e.g., correlation) exists between two genes across the available set of gene expression data. The presence of statistically significant, pairwise statistical association suggests the potential for a biological relationship between the pair of genes, such that changes in the expression levels of one gene correspond to changes in the expression level of another gene. For example, in operon prediction and validation, computing pairwise correlations across all available gene expression data is used to suggest or validate whether contiguous pairs of same-strand genes are in the same operon (Sabatti et al., 2002; Bockhorst et al., 2003; Yeung et al., 2004; Price et al., 2005; Westover et al., 2005; Dam et al., 2007; Okuda et al., 2007; Tran et al., 2007; Wang et al., 2007; Brinza et al., 2010; Ten Broeke-Smits et al., 2010). Similarly, in regulatory network inference algorithms, it is standard to first compute pairwise gene association using a correlation measure (e.g., Pearson correlation, Spearman correlation, mutual information, among others) on a large repository of gene expression data; strong pairwise gene association measures are suggestive that two genes are either co-regulated or act as a transcription factor-target pair in the regulatory network (Margolin et al., 2006; Faith et al., 2007; Kaleta et al., 2010; Mahdi et al., 2012). Yet another use of pairwise association measures in gene expression data analysis is when k-means, principal components or other clustering algorithms are used to suggest sets of genes that show co-regulation (D'haeseleer, 2005; Ringnér, 2008). Recently, pairwise gene correlations have been used to evaluate the quality of external data sets providing biological conjectures (Tintle et al., 2012). Lastly, pairwise correlation between genes is often used in heatmaps of biologically related genes to explore and suggest potential regulatory relationships (Ravcheev et al., 2011) or in integrated metabolic-regulatory models (Chandrasekaran and Price, 2010).

As repositories of gene expression data grow larger and larger, there is a temptation to think that analyses using these larger repositories will automatically generate improved estimates of pairwise gene correlation, and thus downstream analytic methods will yield improved inference about regulatory relationships in bacterial genomes. This argument is based on the idea that larger sample sizes reduce the margin of error of resulting statistical estimates: a well-known statistical fact. For example, a conservative estimate of the margin of error for the pairwise gene-gene Pearson correlation reduces from 0.25 to 0.12 as the sample size increases from 50 samples to 300. However, this margin of error improvement is only true if the underlying parameter being estimated does not change as the sample size increases. However, this is not necessarily the case; the addition of gene expression samples may change the underlying correlation parameter being estimated.

Previous research has emphasized the need to exclude genes showing non-statistically significant changes in gene expression levels between two experiments (differential expression) (Townsend, 2003; Scholtens and Von Heydebreck, 2005) or differential changes below a particular level (Townsend, 2004; Clark and Townsend, 2007), since measurements based on these genes may be mainly noise. Thus, in sets of gene expression data where gene measurements are noisy, correlation estimates will presumably be different than in sets of gene expression measurements are not noisy. Given the well-known relationship between genetic and environmental perturbations and estimates of gene expression levels (True et al., 2014), this suggests that the genetic and environmental perturbations present in a set of expression data may be more important at providing accurate estimates of gene-gene correlation, than simply the amount of samples in the set.

In this paper, we investigate this hypothesis, starting by documenting that there is substantial variability in estimates of pairwise gene correlations for the same pairs of genes, across different repositories of gene expression data and for a diverse set of six bacteria—much more variability than can be explained by chance alone. We then go on to articulate a likely cause of this variability: sampling bias. In particular, the choice of experimental conditions in a repository of gene expression data has substantial impact on estimates of pairwise gene correlation. Having isolated the cause of this variability, we propose a method of mining gene expression data that limits the impact of sampling bias on measures of statistical association, potentially improving downstream analyses which rely on measures of pairwise gene association.

## Methods

### Data

The sources and processing of gene expression data used in the analyses reported here are detailed elsewhere (Tintle et al., 2012). We provide a brief overview here. We focus on six diverse bacteria with 2782 total microarray samples representing mainly unique experimental conditions. In order to ensure robust estimates of pairwise gene correlation, we consider only the 6 bacteria from Tintle et al. (2012), for which at least 150 samples are available. Raw data from Affymetrix CEL files^1^ were consistently normalized using RMA (Irizarry et al., 2003). The number of samples for each organism varied from 176 to 907. Column 2 of Table 1 indicates the total number of samples for each of the six bacteria.

**Table 1:** **Full compendia size and number of partial compendia for each bacteria genome in the analysis**.

**Bacteria**	**Total number of samples in the full compendium for each organism**	**Number of partial compendia (subsets of samples) for each organism (number of samples in each partial compendium)**
*Bradyrhizobium japonicum*	195	3 (A = 83, B = 60, C = 52)
*Escherichia coli*	907	5 (A = 331, B = 212, C = 137, D = 133, E = 94)
*Pseudomonas aeruginosa*	176	3 (A = 70, B = 54, C = 52)
*Shewanella oneidensis*	245	4 (A = 72, B = 61, C = 59, D = 53)
*Staphylococcus aureus*	852	5 (A = 263, B = 228, C = 193, D = 90, E = 78)
*Thermus thermophilus*	407	3 (A = 222, B = 99, C = 86)
Total	2782 samples	23 partial compendia

### Creating partial compendia

We call the total set of samples for which expression data is available the *full compendium* of gene expression data for an organism. For our analyses, we also created *partial compendia* for each organism which represent purposefully created partitions of the full compendium. Each partial compendium is a moderately sized (at least 50 samples) repository of gene expression data for a particular organism, representing a diverse set of experimental conditions. Partial compendia were created to act as stand-ins for independent repositories of gene expression data. As shown in Table 1, Column 3, partial compendia are named with sequential letters of the alphabet, with *A* indicating the largest partial compendia for the organism, *B* the second largest, etc.

We now describe how partial compendia were created. Because gene expression data is typically gathered in related sets of samples, each partial compendium represents a random synthesis of related sets of samples, combined until there are 50 or more samples in the set. For example, data from GEO (*B. japonicum*, *P. aeruginsosa*, *T. thermophilus*) are stored in Gene Expression Omnibus Series (GSEs), representing either a related set of testable hypotheses, samples obtained by the same experimenter or other related factors. To create partial compendia for these three organisms, we randomly combined GSEs until there were 50 or more samples in each partial compendium. For example, for *B. japonicum* there are 71 samples (denoted in GEO as GSMs) in GSE8478 (genomewide transcript analysis of *B. japonicum* bacteroids in soybean root nodules)^2^ and 12 samples in GSE8580 (response of *B. japonicum* wild type and mutant strains to genistein)^3^. We combined these two GSEs to create a partial compendium of 83 samples as indicated in Table 1. We then combined other GSEs to create the rest of the other partial compendia.

A similar approach was taken for *E. coli*, *S. oneidensis* and *S. aureus*. For example, *E. coli* data was obtained from M3D^4^. M3D has collected data from GEO, as well as data deposited directly to M3D by other labs. To create partial compendia for *E. coli* we created a single partial compendium for all GEO data that is in M3D. We then followed a procedure similar to the one detailed in the previous paragraph by randomly combining related sets of samples (e.g., all of the data deposited by a single lab) until at least 50 samples were in the partial compendia. A similar approach was used for both *S. oneidensis* and *S. aureus*.

### Operons and metabolic pathways

In part of our analysis we consider how correlation estimates vary across different partial compendia for pairs of genes that are predicted to be co-regulated by external databases. In particular, we used operon predictions as made by Microbes Online (Price et al., 2005) and metabolic pathway definitions from the SEED (DeJongh et al., 2007).

### Statistical analysis

For each pair of genes, based on the samples in each compendium (full and partial), we computed three different measures of pairwise gene association for all possible pairs of genes: Pearson correlation, Spearman correlation and mutual information. We used R/Bioconductor^5^ to compute all three measures of association. In particular, we used the cor() function to compute the Pearson and Spearman correlations and the mutualInfo() function in the package bioDist to compute mutual information.

To provide an evaluation of the consistency of correlation metrics obtained for the same pair of genes across partial compendia, we used 95% bootstrap confidence intervals, which we computed as described in the remainder of this paragraph. Let *r^k^_i,j_* represent the correlation between genes *i* and *j* in partial compendia *k*. Let *r^k^_i,j,z_* represent the correlation between genes *i* and *j* in the *z^th^* bootstrap set of samples, *z* = 1, …, 1000, from partial compendia *k*. We compute the endpoints of a 95% bootstrap confidence interval on the difference in pairwise gene association measures between partial compendia *k* and *l* by taking the 2.5 and 97.5 percentile of *d* = (*d*^*k,l*^_*i,j*,1_ …,*d*^*k,l*^_*i,j*,1000_), where *d^k,l^_i,j,z_* = *r^k^_r,j,z_* − *r^l^_i,j,z_*. We computed 95% bootstrap confidence intervals using this approach for all pairs of partial compendia for each organism, and separately for each of the three correlation metrics (Pearson correlation, Spearman correlation and mutual information). If the correlations obtained from the different partial compendia are estimating the same, unknown, parameter (the “true” measure of correlation), then, on average, only 5% of the bootstrap confidence intervals obtained from this approach will not contain zero. We note that the “true” measure of correlation being estimated may be any value between -1 and 1, including zero. Furthermore, this true measure correlation may represent either direct, indirect or no co-regulation. Lastly, we note that use of the bootstrap approach explicitly controls for differences in the sizes of the partial compendia, allowing for comparison of partial compendia of any size.

As part of our analysis, we classify gene expression levels as coming from a gene in an “on” state or an “off” state (two state model) using the following procedure. For each gene, we modeled the observed expression distribution across the full compendium as a mixture of an unknown number, *m*, of unequal-variance Gaussian distributions using the R package Mclust^5^. We then found the value of the Bayesian information criterion for each value of *m* (*BIC_m_*) to determine the optimal number of clusters, *m_O_*. In particular, $m_{o}=\{\begin{array}{l} 2,If\;BIC_{2}>(BIC_{2}-10)\;\forall \;i\neq 2\\ \;i,otherwise \end{array}$. In other words, we assume that genes fit the two state model unless strong statistical evidence (change in *BIC* larger than 10) exists to the contrary.

## Results

### Motivating example

We begin by motivating our analysis and approach via a specific example. Consider the following pair of *E. coli* genes: *lexA* (b4043) and *dinF* (b4044), which the literature has strongly suggested are part of the same operon (Krueger et al., 1983; Wade and Struhl, 2004). This operonal relationship is asserted not only in *E. coli* but in a host of other bacterial clades as well (Mazón et al., 2004). Functionally, *lexA* represses the transcription of many genes involved in cellular responses to DNA damage or inhibition of DNA replication, and *dinF* encodes a member of the multidrug and toxic compound extrusion (MATE) family of multidrug efflux transporters (Keseler et al., 2011). In addition to physical mapping results and a functional link consistent with *dinF* and l*exA* being in the same transcriptional unit (Krueger et al., 1983), *lexA* is a gene located only 19 bp away on the same strand. Major databases of operons agree that *lexA* and *dinF* are in the same operon (Price et al., 2005; Keseler et al., 2011; Okuda and Yoshizawa, 2011).

**Figure 1:**
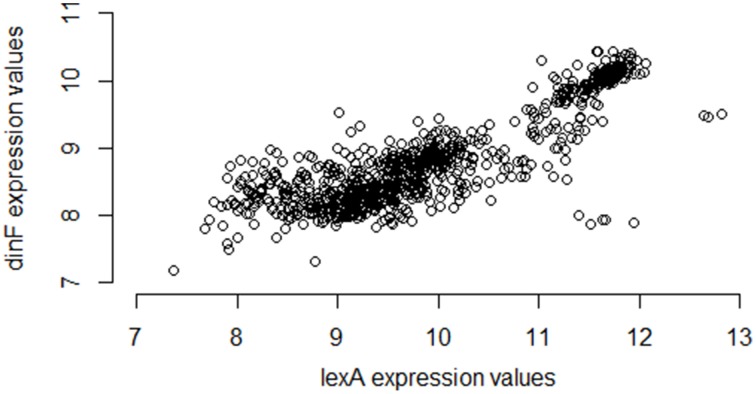
**Expression values for** ***lexA*** **and** ***dinF*** **across 907 microarray samples in a publicly available repository**. The genes *lexA* and *dinF* show a strong pattern of association in RMA-normalized gene expression across the 907 samples available in the M3D repository (full compendium). In particular, as expression levels of *lexA* increase, expression levels of *dinF* also increase. This strong association is captured by the high values of common statistical measures of association (Pearson correlation = 0.86, Spearman correlation = 0.79, Mutual Information = 0.70).

Using a large repository of 907 separate microarray samples available for *E. coli* (Many Microbe Microarrays Database, M3D^4^), and using a standard normalization strategy (Robust Multi-array Average, RMA; details provided in the Methods), we compute the Pearson correlation of the observed expression levels of *lexA* and *dinF* as 0.86, a value indicating a strong statistical association providing experimental confirmation of operonal relationship. Figure 1 gives the corresponding scatterplot for the two genes across the 907 samples.

**Figure 2:**
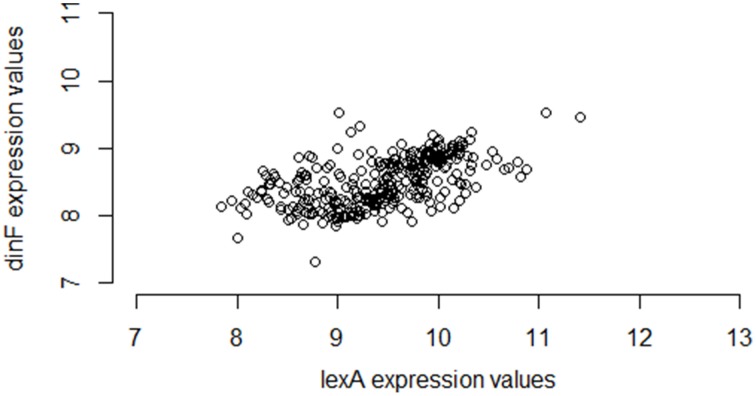
**Expression values for** ***lexA*** **and** ***dinF*** **across 331 microarray samples in a publicly available repository**. The genes *lexA* and *dinF* show a much weaker pattern of association in RMA-normalized gene expression across the 331 samples available in a subset of the M3D repository consisting of gene expression data collected from GEO (28) (partial compendium A). In particular, as expression levels of *lexA* increase, expression levels of *dinF* only modestly increase. This modest association is captured by the modest values of common statistical measures of association (Pearson correlation = 0.56, Spearman correlation = 0.60, Mutual Information = 0.37).

**Figure 3:**
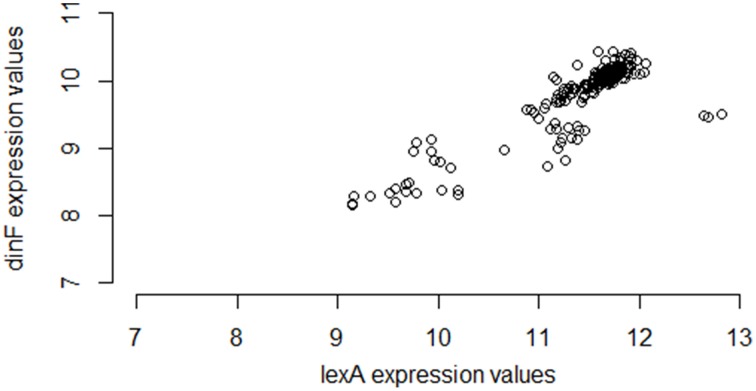
**Expression values for lexA and dinF across 212 microarray samples in a publicly available repository**. The genes *lexA* and *dinF* show a strong pattern of association in RMA-normalized gene expression across these 212 microarray samples (partial compendium B), similar to the pattern seen in the larger set of all 907 samples. In particular, as expression levels of lexA increase, expression levels of dinF increase. This association is captured by the modest values of common statistical measures of association (Pearson correlation = 0.87, Spearman correlation = 0.81, Mutual Information = 0.71).

Interestingly, however, if instead of using M3D, a researcher gathered all 331 available *E. coli* microarray samples from GEO^6^, the observed correlation between *lexA* and *dinF* would appear substantially weaker (Pearson correlation = 0.56; see Figure 2) even when using the same normalization strategies. On the other hand, in another smaller compendium with 212 samples, the correlation is much stronger (Pearson correlation = 0.87; Figure 3) and similar to the larger set of all 907 microarray samples. As we will demonstrate, the differences in correlation estimates (0.56 vs. 0.86 and 0.56 vs. 0.87) are well beyond any difference expected due to random chance alone.

Furthermore, the fact that the entire M3D database has nearly three times as many samples as GEO is not the explanation for the difference in correlation estimates. The smaller set of 212 samples exhibits strong correlation. As we will show, the difference in correlation estimates is due primarily to the experimental conditions of the samples in the set, as opposed to the number of samples.

### Large differences in observed pairwise gene association measures between partial compendia

If pairwise gene association metrics are estimating the same underlying value (“true” correlation) across different partial compendia, then we would expect that, on average, 5% of 95% confidence intervals on the differences will not contain zero. Table 2 provides the proportion of 95% confidence intervals which do not contain zero for each pair of compendia. As can be seen in the table, these proportions are frequently larger than 5% (ranging as high as 97%). The differences are well above 5% across all organisms, all partial compendia and all three association metrics, with the exception of the mutual information metric for *S. oneidensis* which shows only modest inflation. Importantly, the high proportion of confidence intervals which do not include zero is not a result of small compendium sizes. For example, the two largest partial compendia of expression samples for *E. coli* (A and B) have one of the highest proportions of differences. Furthermore, note that differences in compendia sizes are implicitly accounted for in the computation of the bootstrap confidence interval, which will yield wider confidence intervals for small sample sizes.

**Table 2:** **Proportion of 95% bootstrap confidence intervals that do not include zero, comparing partial compendia**.

***B. japonicum***	***E. coli***	***P. aeruginosa***	***S. oneidensis***	***S. aureus***	***T. thermophiles***
AB (46%, 35%, 32%) AC (46%, 44%, 21%) BC (51%, 48%, 13%)	AB (67%, 69%, 55%) AC (37%, 38%, 64%) AD (44%, 43%, 57%) AE (39%, 37%, 94%) BC (60%, 63%, 29%) BD (56%, 59%, 23%) BE (57%, 59%, 60%) CD (37%, 42%, 11%) CE (38%, 37%, 30%) DE (45%, 43%, 40%)	AB (25%, 22%, 5%) AC (32%, 30%, 8%) BC (25%, 21%, 6%)	AB (38%, 37%, 8%) AC (39%, 37%, 10%) AD (42%, 40%, 6%) BC (32%, 29%, 2%) BD (37%, 37%, 8%) CD (42%, 44%, 10%)	AB (40%, 44%, 13%) AC (38%, 42%, 17%) AD (64%, 62%, 90%) AE (32%, 34%, 88%) BC (29%, 27%, 10%) BD (63%, 63%, 91%) BE (29%, 29%, 89%) CD (64%, 63%, 86%) CE (32%, 33%, 79%) DE (62%, 59%, 13%)	AB (40%, 39%, 67%) AC (35%, 30%, 97%) BC (48%, 43%, 20%)

*For each organism, each pair of partial compendia are compared and the percent of Pearson correlation, Spearman correlation and mutual information confidence intervals that don't include zero are provided. If the partial compendia are estimating the same underlying parameter, as we would expect and desire, on average, only 5% of the confidence intervals will not include 0. However, in almost all cases substantially more than 5% of the confidence intervals do not include zero suggesting that for many genes the different partial compendia are estimating different parameters, regardless of the gene association metric being used*.

**Figure 4:**
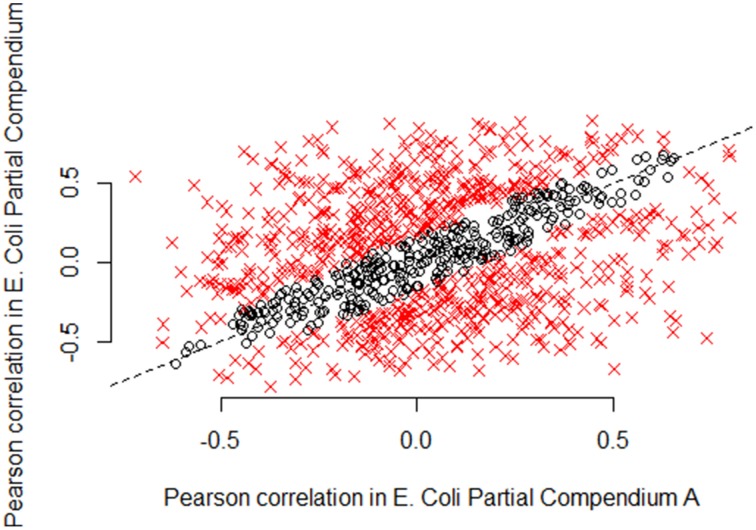
**Pearson correlations according to partial compendia A and B in 1000 random** ***E. coli*** **gene pairs**. For large compendia (as we use here: 331 samples and 212 samples, respectively) we expect limited variability in gene expression correlation measures between the compendia. In particular, we expect most correlations to be close to the line y = x, if the same underlying parameter is being estimated. We computed 95% confidence intervals on the difference in correlation estimates for 1000 random *E. coli* gene pairs. Black circles indicate pairs of genes for which the correlation estimates are similar (95% confidence interval includes zero), while red x's indicate gene pairs for which the correlation estimates are not similar (95% confidence interval does not include zero). As shown in the figure by the preponderance of red x's, the majority of pairwise correlation values are well outside the expected range, representing substantially more variability than is expected due to chance alone. The Pearson correlation computed on the scatterplot shown in this figure, is only 0.33, representing very weak association between the Pearson correlation of pairs of genes in partial compendia A and B for *E. coli*.

**Figure 5:**
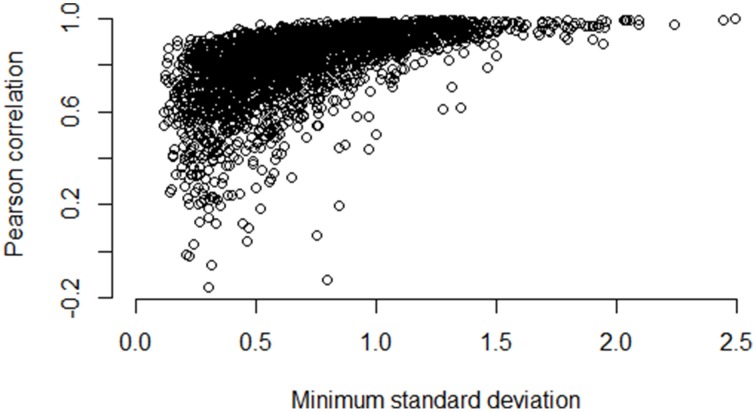
**Pairwise Pearson correlation versus minimum standard deviation of gene expression value: operons**. As the minimum standard deviation of the gene pair increases, the correlation between genes likely to be in the same operon also increases. The Pearson correlation for the scatterplot is 0.58.

**Figure 6:**
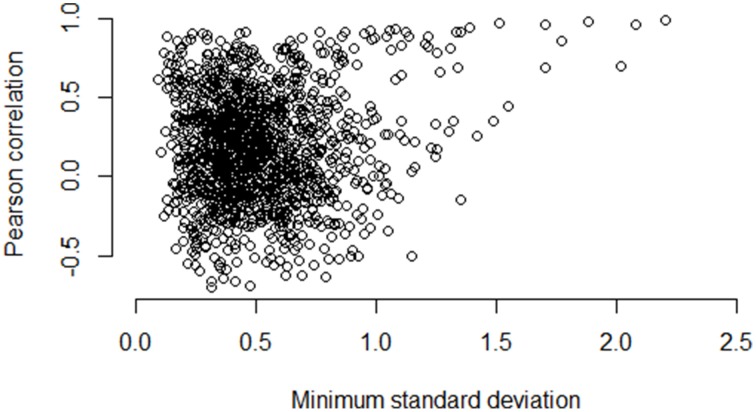
**Pairwise Pearson correlation versus minimum standard deviation of gene expression value: non-operons**. As the minimum standard deviation of the gene pair increases, the correlation between genes likely to not be operons shows little discernible pattern also increases. The Pearson correlation for the scatterplot is 0.10.

To more fully understand the issue, we focus on *E. coli* partial compendia A and B. Figures 4–**6** plot the actual values of the Pearson, Spearman and mutual information for 1000 randomly chosen pairs of genes for *E. coli* for partial compendium A and partial compendium B. If these two subsets of samples were generally providing estimates of correlation that were estimating the same underlying value we would expect the majority of points to fall near the line *y* = *x*. In fact, for Pearson correlation, statistical theory says, conservatively, that at least 95% of the values should fall within 0.14 of the line y = x (Figure 4, blue dashed lines). However, the vast majority of points are well outside of this range. A similar lack of consistency between metrics across the two compendia is observed for both the Spearman correlation (Supplemental Figure 1) and mutual information (Supplemental Figure 2).

We consider two approaches to predict pairwise gene correlation values. The naïve approach, as considered in the previous paragraphs, says that the correlation value for a given pair of genes, *i* and *j*, in partial compendium *k*, *r^k^_i,j_*, should be well predicted by the correlation value for the same gene pair in partial compendia *l*, *r^l^_i,j_*. An uninformative approach would be to predict *r^k^_i,j_* based on a randomly selected gene pair (*y,z*; *y,z* not equal to *i,j*) in partial compendia *l*, *r^l^_y,z_*. Table 3 illustrates that the uninformative approach outperforms the naïve approach (meaning that $\left|r_{i,\;j}^{k}-r_{i,j}^{l}\right|>\left|r_{i,\;j}^{k}-r_{y,z}^{l}\right|$ ∼30–70% of the time across all organisms and all correlation metrics. Thus, in general, there is little information in the correlation between genes *i* and *j* in compendium *l* useful for prediction of correlation between genes *i* and *j* in compendium *k*.

**Table 3:** **Predicting pairwise gene correlations using a plausibly informative vs. uninformative approach**.

**Bacteria**	**Percent of times that uninformative approach outperformed naïve approach**		
	**Pearson correlation**	**Spearman correlation**	**Mutual information**
*Bradyrhizobium japonicum*	37.4%	37.1%	49.9%
*Escherichia coli*	40.7%	40.6%	60.7%
*Pseudomonas aeruginosa*	32.9%	34.3%	45.8%
*Shewanella oneidensis*	40.6%	42.5%	50.4%
*Staphylococcus aureus*	34.2%	36.2%	65.2%
*Thermus thermophilus*	27.8%	28.3%	67.5%

*The naïve approach uses the pairwise gene correlation value in partial compendia l, to predict the value for the same pair of genes in partial compendia k, the uninformative approach uses a random value to make the prediction. If the naïve approach were no better than the uninformative approach, we would expect the values in the table to be ~50%. Since the values in the table are quite close to 50%, there is a high degree of noise in the correlation estimates and limited signal. If the values were close to 0, the naïve approach would, typically, be outperforming the uninformative approach*.

### Understanding why the large differences in correlation estimates exist

To begin to understand why the large differences in correlation exist between different pairs of partial compendia, we return to the example of the *lexA*/*dinF* operon for *E. coli* described earlier (Results: Motivating Example). Figure 1 suggests that *lexA* and *dinF* tend to be either “on” (gene expression levels above ~11 for *lexA* and above ~9.5 for *dinF*) or “off” (gene expression levels less than ~11 for *lexA* and less than ~9.5 for *dinF*) concurrently. Table 4 provides the values of the three association metrics for each of the five partial compendia and the full compendium, along with the percent of samples in the compendium for which the expression values of either just *lexA*, or both *lexA* and *dinF* illustrate an “on” state as defined earlier in this paragraph. Furthermore, Table 4 provides the standard deviation of the absolute expression values for each gene in each compendium.

**Table 4:** **Values of the association metrics for the *lexA-dinF* operon overall and in different partial compendia**.

	**Full compendium**	**Partial compendium A**	**Partial compendium B**	**Partial compendium C**	**Partial compendium D**	**Partial compendium E**
Pearson	0.86	0.56	0.87	0.40	0.92	0.34
Spearman	0.79	0.60	0.81	0.37	0.92	0.23
Mutual Information	0.70	0.37	0.71	0.43	0.99	0.35
Percent of samples in which the expression level of lexA is “on” (above 11)	24% (216/907)	1% (2/331)	88% (186/212)	8% (11/137)	13% (17/133)	0% (0/94)
Percent of samples in which the expression level of lexA is “on” (above 11) and the expression level of dinF is “on” (above 9.5)	20% (185/907)	0% (1/331)	80% (169/212)	2% (3/137)	9% (12/133)	0% (0/94)
Standard deviation of absolute expression for lexA	1.13	0.62	0.64	0.99	0.92	0.55
Standard deviation of absolute expression for dinF	0.71	0.36	0.53	0.47	0.55	0.26

This pair of genes, like most (see previous section for details), shows substantial differences in the estimated correlation among the five partial compendia. In particular, partial compendia B and D show strong correlation (as is expected since this a known operon) while partial compendia A, C, and E show weak to moderate correlation. We gain insight into why the values of the association metrics are different in compendia B and D as compared to A, C, and E by examining the percent of samples in each partial compendia for which the gene (or gene pair) are “on.” In particular, we note that in both partial compendia B and D there is at least a 10/90 split of “on” and “off” samples, whereas in compendia A, C and E the genes are rarely on. Referring back to Figure 1, compendia A, C, and E can be thought of as possessing samples which *only* occur in the lower left “cluster,” while compendia B and D have at least 10% of their samples from each of the two clusters. We note that in the case of partial compendium C, while there is a modest percentage (8%) of samples for which the *lexA* gene is “on,” 8 of these samples have low levels of the *dinF* gene (less than 9.5), which, ultimately, yields a low correlation. This is further underscored by noting that the *lexA* and *dinF* genes are both “on” in only 2% of samples in compendium C. Thus, unless the percent of samples for which both of the genes are on (and off) is above 10%, the estimate of the pairwise association is questionable for the *lexA* and *dinF* operon. If we view the variability within the off (or on) state as “noise” and the change in states as “signal,” then it is clear that if samples come from primarily within the off (or on) state, we have only “noise” and the “true signal” (pairwise gene association) is not present. Conversely, if we obtain an adequate number of samples within both states, the samples will provide sufficient signal to rise above the noise and, ultimately, we will observe a strong pairwise gene association. Statistically, this issue is called sampling bias: some compendia present a biased view of true correlation.

This on-off sampling argument is not limited to the *lexA-dinF* operon in *E. coli*; in fact it is widespread across all organisms and operons or metabolic pathways in our analysis. To conduct a more comprehensive analysis, we consider three types of gene pairs across all six bacteria. First, we consider contiguous gene pairs which have a posterior probability of at least 99% of being an operon according to MicrobesOnline (Price et al., 2005), which uses primarily genomic evidence (e.g., distance between genes, conservation across organisms, related function) to make its predictions. Thus, we take a posterior probability of at least 99% as suggesting that the pair of genes is in fact an operon, thus, we should observe large (strong) correlations between pairs of genes in this group. Second, we consider contiguous gene pairs with posterior probability of being in an operon of at most 1% according to MicrobesOnline. Here, weaker correlation in the expression data is expected. While we recognize that not being in an operon does not necessarily mean that the genes will not be correlated, we can reasonably anticipate that the gene pair will show low or no correlation in many of these cases. Lastly, we consider pairs of genes which are located in the same metabolic pathway (Tintle et al., 2012). As with operons, we expect genes in the same pathway to have a strong pairwise association in gene expression data though we do not expect the true correlation to be as strong as it is for operons.

Table 5 provides the average Pearson correlation of pairs of genes classified by the biological source of the gene pair. This analysis was conducted using the full compendium of samples for each organism, using genes for which a two-state clustering strategy to classify genes as “on” or “off” is valid (see Methods for details). The percent of on (or off) categorization is based on the minimum on or off percent across the gene pair. So, a percent close to one half means that both genes are sampled approximately equally from both the “on” and the “off” state across the set of samples in the partial compendia being compared, while a percent close to zero means that at least one gene is almost always on or almost always off in the samples in the full compendium.

**Table 5:** **Average Pearson correlation across all pairs of genes, cross-classified by biological grouping and percent on/off**.

**Minimum percent of samples in the compendia for which the genes in the pair are on (or off)^a^**	**Variability in expression levels** **Less** 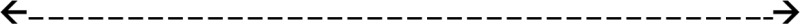 **More**					
	**Less than 5%**	**5–10%**	**10–20%**	**20–30%**	**30–40%**	**40–50%**
Likely operons^b^	0.72 (294)	0.75 (320)	0.81 (610)	0.84 (493)	0.87 (310)	0.88 (123)
Likely non-operons^c^	0.20 (254)	0.17 (238)	0.16 (186)	0.21 (186)	0.19 (86)	0.06 (28)
Gene pairs in same pathway^d^	0.23 (1128)	0.20 (1280)	0.28 (1994)	0.38 (1779)	0.35 (711)	0.47 (266)

*In parentheses is the number of gene pairs satisfying the conditions to be included in each cell. The full compendium of expression data is considered*.

a *The minimum of the percent of samples arrays for which either gene is on or off in the full compendium of samples*.

b *Posterior probability of being in an operon based on genomic evidence is more than 99% according to MicrobesOnline (Bockhorst et al., 2003)*.

c *Posterior probability of being in an operon based on genomic evidence is less than 1% according to MicrobesOnline (Bockhorst et al., 2003)*.

d *Pathway definitions provided by the SEED (Tintle et al., 2012)*.

The results in Table 5 demonstrate that the intuition described above is correct. In particular, correlation estimates for pairs of genes from sets of samples which do not substantially or frequently perturb the genes' expression levels (e.g., change on-off states), show less consistency with *a priori* biological prediction than correlation estimates obtained from sets of compendia with a substantial number of samples from both on and off states. In particular, for pairs of genes likely to be in the same operon, as the variability in expression levels increases the average Pearson correlation also increases. This pattern is also observed for pairs of genes in the same metabolic pathway though, expectedly, the absolute value of the correlations is not as strong. Furthermore, for pairs of genes likely not in the same operon, as the variability in expression increases, the average Pearson correlation does not increase. The Spearman correlation and mutual information show similar patterns (details not shown). Thus, we see increasing correspondence between *a priori* biological knowledge and empirical evidence from transcriptional data as we increase the variability in expression levels. Statistically speaking, sampling from both states is a stratified sampling strategy which means less bias in downstream estimates of pairwise gene association.

While on-off classification provides a convenient dichotomy to simplify our description of the problem, we also consider a more general approach which will work for all gene pairs (Note: 17.45% of all gene pairs were eliminated from the analysis shown in Table 5 because one or both genes did not fit the two-state (on-off) model; see Methods for details). Referring back to Table 4, we see that the lexA-dinF operon shows less variability (smaller standard deviation) in partial compendia A, C, and E—exactly those compendia discussed earlier as providing suspicious estimates of the correlation.

Going further and generalizing the result from Table 5, we predict that, in general, genes with more variability in gene expression values (e.g., larger standard deviation in expression levels) are more likely to be changing in a biologically meaningful way (not simply due to measurement error or other random variation), whereas genes with less variability in gene expression values are less likely to be changing values due to underlying biological reasons—more likely due to chance variation. Thus, we consider the standard deviation as an alternative approach to accurately predict whether, for a given gene pair and set of samples, the researcher can have confidence in the correlation estimate obtained.

**Figure 7:**
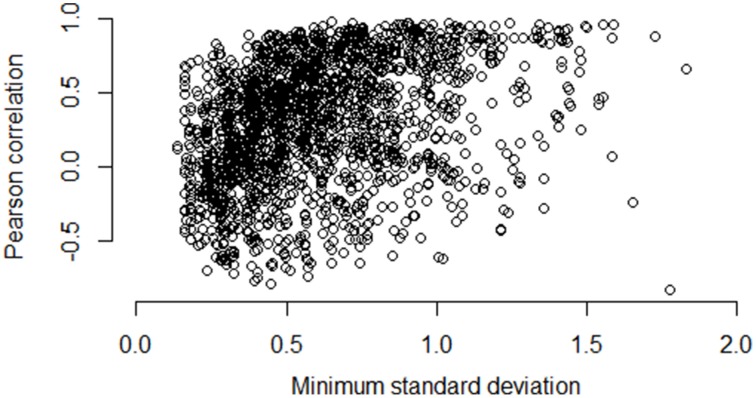
**Pairwise Pearson correlation versus minimum standard deviation of gene expression value: pathways**. As the minimum standard deviation of the gene pair increases, the correlation between genes in the same pathway shows a generally increasing pattern. The Pearson correlation for the scatterplot is 0.38.

Figures 5–**7** illustrate how the standard deviation associates with the quality of the association metric. In particular, Figure 5 provides a scatterplot of all of the operon pairs from Table 5, plotting the observed Pearson correlation against the minimum standard deviation of the two genes in the pair. Figures 6, 7 are similar, except for non-operon pairs and pathways respectively. Notably, we see a strong association between the standard deviation of the expression values and the value of the Pearson correlation for operons, a weaker association for pathways, and a much weaker, or non-existent, relationship for non-operons. In particular, for low minimum standard deviation, estimated correlations between genes in an operon may be good or bad, but the higher the minimum standard deviation, the more accurate the correlation estimate will be. Similar patterns are seen for both the Spearman correlation and mutual information (details not shown).

### Controlling sampling bias at a genome-wide level when mining large repositories of expression data

Our analysis has shown that bias in correlation estimates based on large gene expression repositories is both rampant and substantial. Thus in order to control sample bias when mining large repositories of expression data we propose that either the percent on-off or the standard deviation be used to flag potentially biased correlation estimates. In essence, through the flagging approach, we can provide confidence that the correlation estimate for a gene pair is not biased.

In particular, we propose the following when a two-state (on-off) clustering model is reasonable for the observed expression data for a pair of genes: unless both states for both genes are present in at least 10% of the samples, the correlation estimate should be flagged as potentially biased. In all cases, regardless of whether the two-state (on-off) clustering model is reasonable, genes with standard deviations of less than 0.5 for RMA normalized data suggest that downstream correlation estimates may be biased. These “rules of thumb” were derived by exploring the sensitivity and specificity of different standard deviation and percent on-off rules. Tables 6, 7 provides the sensitivity and specificity of different standard deviation and percent of sample cutoffs at identifying pairs of genes with correlation estimates that are likely to be biased estimates of the true correlation. In particular, for pairs of genes predicted by MicrobesOnline to be highly likely in the same operon (predicted probability of at least 0.99), we examined how often different rules of thumb “flagged” Pearson correlations above or below 0.6. We used 0.6 as a threshold of meaningful correlation for genes truly in an operon, though other values are possible. A good rule of thumb should flag most genes in operons with correlations below 0.6 as biased, while not flagging many operon genes with correlation above 0.6. As shown in Tables 6, 7, a standard deviation of 0.5 and having both states present in at least 10% of the samples tended to provide maximal values of sensitivity + specificity. We note that, as with any rules of thumb, sensitivity can be improved by increasing the standard deviation threshold used to flag gene pairs (e.g., standard deviation cutoff larger than 0.5), but at the expense of specificity and vice versa.

**Table 6:** **Sensitivity and specificity of different standard deviation cutoffs**.

Standard deviation	0.1	0.3	0.5	0.7	0.9
Sensitivity	0 (0/287)	0.36 (103/287)	0.77 (221/287)	0.95 (274/287)	0.99 (283/287)
Specificity	1 (2448/2448)	0.93 (2272/2448)	0.72 (1772/2448)	0.49 (1193/2448)	0.31 (753/2448)
Sensitivity+ Specificity	1	1.29	1.49	1.44	1.3

*For 2735 pairs of genes likely to be in the same operon, 287 have correlations below 0.6 suggesting that these correlation estimates are substantially biased, since the true correlation between two genes in the same operon should be close to 1. Sensitivity is computed by calculating the number of these pairs of genes with standard deviations below a certain threshold. 77% (221/287) of the 287 pairs of operon pairs with low correlation also have low (<0.5) standard deviation. Of the operon pairs with larger correlations (0.6), nearly three-fourths (72%) have standard deviations above 0.5*.

**Table 7:** **Sensitivity and specificity of different state cutoff rules**.

Minimum represented cutoff	0.025	0.05	0.1	0.2
Sensitivity	0.11 (26/244)	0.25 (61/244)	0.51 (125/244)	0.77 (188/244)
Specificity	0.95 (1938/2042)	0.87 (1782/2042)	0.74 (1511/2042)	0.45 (915/2034)
Sensitivity + specificity	1.06	1.12	1.25	1.22

*For 2286 pairs of genes likely to be in the same operon and for whom the two state model is a good fit, 244 have correlations below 0.6 suggesting that these correlation estimates are substantially biased, since the true correlation between two genes in the same operon should be close to 1. Sensitivity is computed by calculating the number of these 244 low correlation pairs with the percent of samples in a state below a certain threshold. 51% (125/244) of the 244 low correlation pairs also have less than 10% of the experiments classified to one of two states (on-off). Of the operon pairs with larger correlations (0.6), nearly three-fourths (74%) have at least 10% of the experiments in each of the two states*.

Application of the standard deviation rule of thumb to all 2735 pairs of genes likely to be in the same operon (Microbes Online predicted probability of at least 0.99), yielded 287 gene pairs with Pearson correlation less than 0.6 in the full compendia, 221 of which had standard deviation less than 0.5; a sensitivity of 77% (221/287). Specificity, was also high (72%; 1772/2448). A similar approach applied to gene pairs for the two state model, the 10% rule yields sensitivity of 51% and specificity of 74%. More details are in Tables 6, 7.

In a brief follow-up analysis, we confirmed that the strategy of eliminating genes with low variability genes does not merely eliminate genes with low average absolute expression values. We note that across the 27,140 genes in our analysis, 25% of the genes with standard deviation less than 0.5 have average expression values above 8 (ranging as high as 14.9). Similarly, among the genes for which a two state model fits the data well, 25% of the genes with less than 10% of the experiments in one of the two states have a mean expression of 8.6 or larger.

## Discussion

In this manuscript we have documented widespread and substantial bias in correlation estimates obtained from large repositories of bacterial gene expression data. At a minimum, this bias leads to unclear downstream inference about the biology of bacterial organisms while, at its worst, this bias can lead to completely incorrect inferences based on the correlation metrics. We have demonstrated that this bias is present across a diverse set of organisms, correlation metrics and gene pairs. Importantly, we have clearly shown that the problem is not simply solved by adding more samples to the repository.

Statistically speaking, the issue is sampling bias. In particular, for a given pair of genes it is often the case that in any particular repository of gene expression data, both genes in the pair have not been perturbed (e.g., environmentally or genetically) in order to cause the meaningful change in expression level necessary in order to yield unbiased estimates of gene-gene association. For genes where a two-state (on-off) model is a reasonable abstraction of the data, sampling bias is easily described as failing to sufficiently (if at all) sample one of the two states, for at least one of the genes. For genes which show a more continuous change in expression level, sampling bias means failing to include samples which have substantially changed the expression level of the gene.

We have proposed a method which can flag correlations which may be particularly prone to sampling bias. In particular, we identify pairs of genes where, for at least one of the genes, less than 10% of the samples are from one of the two (on-off) states, or where one of the genes has a standard deviation less than 0.5. An application of our method to pairs of genes that are highly likely to be in operons reduced the number of low correlations and provided reasonable levels of sensitivity and specificity. Since operons can be viewed as close to a gold standard of pairs of genes that should be correlated, the method may be widely applicable to any gene pair being considered biologically. A handful of minor limitations of the method are worth noting. First, the partial compendia in our analysis ranged from ~50 to 300 samples and so, for sets of samples containing less than 50 or more than 300 samples, the 10% rule of thumb may not be appropriate. Caution should be exercised when estimating gene-gene correlation on sets of less than 50 samples as correlation estimates can illustrate substantial chance variability in these contexts, even if sampling bias is not present (for example, a margin of error of over 0.25 when estimating gene-gene correlations using the Pearson correlation is possible in these contexts). Secondly, the 0.5 standard deviation rule is based on RMA-normalized data and may not apply for other normalization strategies (e.g., MAS 5.0, MBEI). However, since most expression measures ultimately log-transform the data, this rule may be robust to alternative normalization strategies. Use of gene pairs likely to be in operons to develop appropriate thresholds for use with other technologies (e.g., RNAseq), is possible by following a process similar to the one we conducted here. Relatedly, our analysis only considered three possible, though commonly used, measures of association between genes and further work is needed to extend the result to alternative measures. We note that other flagging strategies exist to identify genes that are never activated in gene expression data, or never show significant change between conditions (e.g., the gene shows know statistically significant fold change between any two conditions, Townsend and Hartl, 2002; Faith et al., 2007; Zhang et al., 2010). Further work is needed to evaluate the performance of different flagging strategies with regards to the sensitivity/specificity at providing accurate pairwise gene-gene correlation estimations. Additional work is also needed to explore novel, more complex flagging strategies, which would ideally improve sensitivity and specificity and use confidence, instead of a simple flag, to better quantify evidence of bias. Finally, we note that while our approach here is to identify estimates prone to bias, we are currently pursuing *in silico* methods of correcting biased estimates. Preliminary efforts suggest some promise to such an approach and are being pursued.

Our use of the synthetically created partial compendia is worth brief discussion. In order to provide a maximally informative analysis and pinpoint the root of the unexplained variability issue, we created partial compendia designed to represent the sorts of “sets of samples” that are commonly used in downstream statistical analyses using pairwise gene correlation metrics. While, in general, these are not actual sets that individuals would use (except in the case of partial compendia A for *E. coli* which consists of all of the GEO samples for *E. coli*), we believe they capture very well what is done in practice to generate large sets of samples. Namely, the samples from many series of experiments are compiled together with little regard for experimental diversity. The typical goal is simply to get “enough samples” in order to yield robust estimates of the correlation. Thus, a minimum of 50 samples is typically considered sufficient to compute pairwise gene correlations, with the generally accepted belief that when it comes to repositories “the bigger the better.” Thus, while artificial, we feel that partial compendia illustrate precisely how repositories are constructed in practice. Furthermore, while we used partial compendia to illustrate the extent of the problem, it is worth noting that we conducted analyses on the full compendia, which could be viewed as all available samples for these organisms; the problems we've identified are not merely limited to the partial compendia, as we illustrated in our analysis of the full compendia in conjunction with the operon and pathway data.

We also note that the current “bigger is better” philosophy does have some merit. With additional samples it is certainly more likely that on or off states will be included in the sample. However, the important caveat is that if a researcher adds substantially more samples, and, for a particular pair of genes, the additional samples have little variation in expression levels for the gene pair of interest, then the observed levels of variability for the gene pair will decrease, and, potentially reduce the level of statistical evidence of underlying association (correlation)—in other words, it is possible for the sampling bias to increase with the addition of more samples. This would happen in a case where additional technical or biological replicates of experiments were added. Importantly, in many procedures and papers utilizing pairwise gene correlations from large sets of gene expression data (Westover et al., 2005; Margolin et al., 2006; Faith et al., 2007; Okuda et al., 2007; Chandrasekaran and Price, 2010), there is an implicit assumption that bigger will always be better. Our proposed gene flagging approach limits errors in downstream inference using these methods which results from analysis of genes showing little change in gene activity across the set of experiments being analyzed.

As the field transitions to RNAseq technologies, these problems will not go away. While further study is needed, since RNAseq is merely an alternative way to quantify genome-wide expression levels, in principle these same sampling bias issues, leading to biased estimates of pairwise gene correlation will still exist. Further research is needed to evaluate approaches proposed in this paper, as potentially appropriate for RNAseq samples.

## Conclusions

We have documented sampling bias as a cause of widespread, previously unexplained variability in pairwise gene association metrics between large, diverse sets of gene expression samples. We have proposed a preliminary, but easy to implement, approach which can flag pairwise gene correlations which may be particularly sensitive to sampling bias. Further work is necessary to investigate if there are ways of improving the sensitivity and specificity of the proposed flagging strategy and conducting bias correction to correlation estimates *in silico*. In the meantime, we suggest that gene-gene correlation estimates computed on genes where at least one of the genes has a standard deviation of expression values less than 0.5, be flagged as potentially biased in all analyses which compute gene-gene correlation from large, RMA-normalized expression repositories.

### Conflict of interest statement

The authors declare that the research was conducted in the absence of any commercial or financial relationships that could be construed as a potential conflict of interest.

## Footnotes

^1^http://www.affymetrix.com

^2^http://www.ncbi.nlm.nih.gov/geo/query/acc.cgi?acc=GSE8478

^3^http://www.ncbi.nlm.nih.gov/geo/query/acc.cgi?acc=GSE8580

^4^http://m3d.mssm.edu/

^5^http://www.bioconductor.org

^6^http://www.ncbi.nlm.nih.gov/geo/

## Abbreviations

BIC: Bayesian information criterion
GEO: Gene Expression Omnibus
GSE: Gene Expression Omnibus Series
M3D: Many Microbe Microarrays Database
MATE: Multidrug and toxic compound extrusion
RMA: Robust Multi-array Average.
