# Supplementary material for: Cautions about the reliability of pairwise gene correlations based on expression data
Source: Front Microbiol. 2015 Jun 26;6:650. doi: 10.3389/fmicb.2015.00650 (PMC4481165; doi:10.3389/fmicb.2015.00650)
Supplement: Supplementary file 1 [file DataSheet1.DOCX]

**Supplemental Figure 1 - Spearman correlations according to partial compendia A and B 1000 random *E. coli* gene pairs

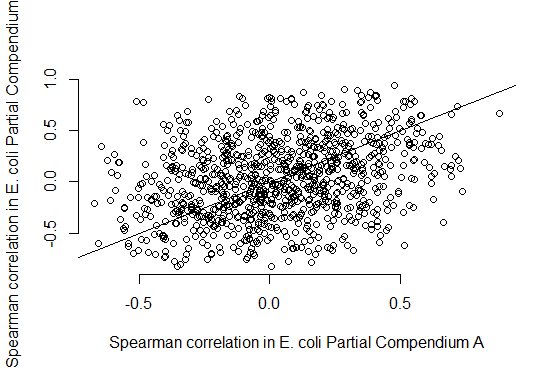
**

We expect limited variability in gene expression correlation measures between compendia. The majority of pairwise correlation values are far away from the line y=x (solid black line). The Pearson correlation computed on the scatterplot shown in Figure 5 is only 0.30, representing very weak association between the Spearman correlation of pairs of genes in partial compendia A and B for *E. coli*.

**Supplemental Figure 2 - Mutual information according to partial compendia A and B 1000 random *E. coli* gene pairs**

**
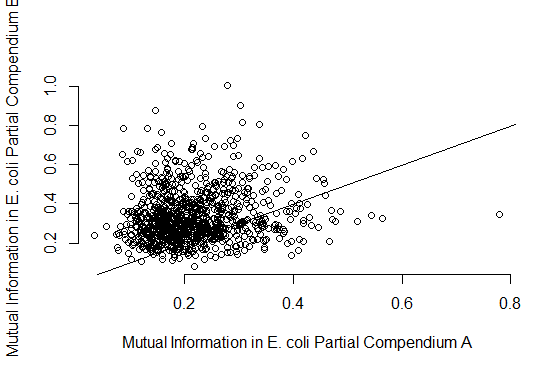
**

We expect limited variability in gene expression correlation measures between large compendia. The majority of pairwise mutual information values are far away from the line y=x (solid black line). The Pearson correlation computed on the scatterplot shown in Figure 6 is only 0.12, representing very weak association between the mutual information of pairs of genes in partial compendia A and B for *E. coli*.
